# Use of Novel Cardanol-Porphyrin Hybrids and Their TiO_2_-Based Composites for the Photodegradation of 4-Nitrophenol in Water

**DOI:** 10.3390/molecules16075769

**Published:** 2011-07-07

**Authors:** Giuseppe Vasapollo, Giuseppe Mele, Roberta Del Sole, Iolanda Pio, Jun Li, Selma Elaine Mazzetto

**Affiliations:** 1 Department of Engineering for Innovation, University of Salento, Arnesano Street, Lecce 73100, Italy; 2 Key Laboratory of Synthetic and Natural Functional Molecule Chemistry of Ministry of Education, College of Chemistry and Materials Science, Northwest University, Xi’an, Shaanxi 710069, China; Email: junli@nwu.edu.cn (J.L.); 3 Laboratory of Products and Processes Technology (LPT), Department of Organic and Inorganic Chemistry, Federal University of Ceará, Fortaleza 6021, Brazil; Email: selma@ufc.br (S.E.M.)

**Keywords:** cardanol, porphyrins, metalloporphyrins, heterogeneous photocatalysis, 4-nitrophenol, porphyrin-TiO_2_ photocatalysts

## Abstract

Cardanol, a well known hazardous byproduct of the cashew industry, has been used as starting material for the synthesis of useful differently substituted “cardanol-based” porphyrins and their zinc(II), copper(II), cobalt(II) and Fe(III) complexes. Novel composites prepared by impregnation of polycrystalline TiO_2_ powder with an opportune amount of “cardanol-based” porphyrins, which act as sensitizers for the improvement of the photo-catalytic activity of the bare TiO_2_, have been used in the photodegradation in water of 4-nitrophenol (4-NP), which is a toxic and bio-refractory pollutant, dangerous for ecosystems and human health.

## 1. Introduction

Cardanol is a naturally occurring phenol obtained by vacuum distillation of cashew nut shell liquid (CNSL), a waste byproduct obtained in the cashew nut processing industry [1,2,3,4,5]. Despite the fact that cardanol could really be considered a dangerous toxic waste, mainly due to the massive amounts of CNSL produced annually, it represents a precious natural renewable resource which can be used as a starting material for the preparation of a large variety of useful chemicals [6].

In fact, the preparation of fine chemicals from natural and renewable materials is nowadays becoming an attractive topic of research especially for the purpose of recycling huge amounts of agro-industrial waste.

**Figure 1:**
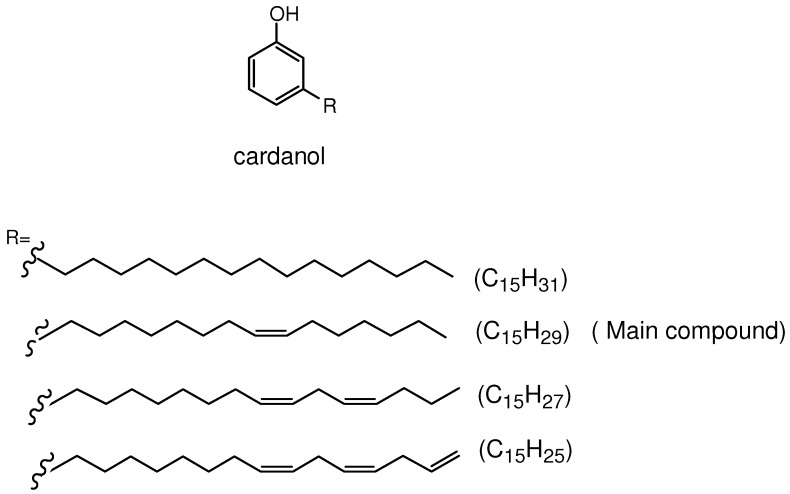
Main components of the cardanol mixture.

The yellow oil obtained by vacuum distillation of CNSL, that for simplicity we call cardanol, contains 3-*n*-pentadecylphenol, 3-(pentadeca-8-enyl)phenol, 3-(pentadeca-8,11-dienyl)phenol, and 3-(pentadeca-8,11,14-trienyl)phenol in approximately 8%, 80%, 8%, 6%, respectively (Figure 1) [7].

On the other hand, the photodegradation of organic pollutants in water is a topic of growing interest and much attention has been devoted in recent years from both academic and industrial researchers to design new photocatalytic systems having effective application in environmentally friendly processes like the TiO_2_-based photocatalysts used for the oxidative degradation of various kinds of organic pollutants [8,9,10].

4-Nitrophenol (4-NP) is a harmful and bio-refractory contaminant which can cause considerable damage to the ecosystem and human health. For this reason its efficient degradation in aqueous effluents is important in order to minimize its deleterious effects as well as environmental problems [11,12,13,14].

In the past, we have used 3-*n*-pentadecylphenol (hydrogenated cardanol), as well as cardanol, as basic materials for the preparation of fine chemicals such as *meso-*tetrasubstituted cardanol-based A_4_-porphyrins [15,16]; but, we noted that only a few examples concerning the use of 3-*n*-pentadecyl- phenol-based porphyrins as sensitizers to enhance the photoactivity of TiO_2_ in the photodegradation of pollutants in water under UV light, have been reported [17].

Therefore, continuing our research in this area, we like to report here the synthesis and characterization of new *meso-*AB_3_ and *trans*-A_2_B_2_ porphyrins, 5,10,15-triphenyl-20-mono-[4-(2-(3-pentadec-8-enyl)phenoxy)ethoxy]phenylporphyrin (**3**) and 5,15-diphenyl-10,20-di-[4-(2-(3-pentadec-8-enyl)phenoxy)ethoxy]phenylporphyrin (**4**), and their metal derivatives (M = Zn, Cu, Co and Fe).

We would also like to report studies concerning the photocatalytic activity of these compounds, once deposited onto TiO_2_, in photodegradation of 4-nitrophenol contained in the water. The advantages related to the use of cardanol-based porphyrins containing double bonds in the cardanol side chain has also been noted in this work.

## 2. Results and Discussion

### 2.1. Synthesis and Characterization of Cardanol Based Porphyrins

**Scheme 1:**
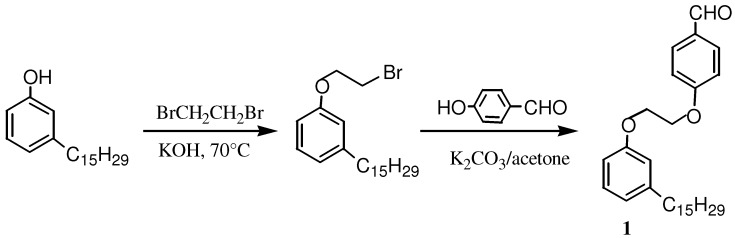
Synthesis of 4-[2-(3-(pentadeca-8-enyl)phenoxy)-ethoxy]-benzaldehyde (**1**).

In this work, the term cardanol is used to refer mainly to 3-(pentadeca-8-enyl)-phenol, the monoolefinic component which can be obtained almost pure from the cardanol oil through distillation and chromatographic separation, the purity of which, enough for our purposes, was confirmed by GC-MS and NMR analyses. The *meso-*AB_3_ and *trans*-A_2_B_2_ porphyrins were obtained, using 4-[2-(3-(pentadeca-8-enyl)phenoxy)-ethoxy]-benzaldehyde (**1**) which was prepared from cardanol through two steps as shown in Scheme 1, following the procedure reported in the literature [6,18].

**Scheme 2:**
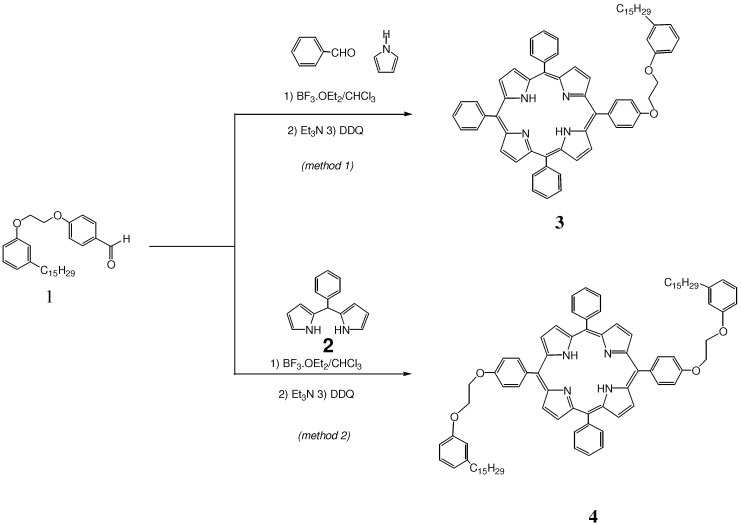
Synthesis of *meso-*AB_3_ and *trans*-A_2_B_2_ cardanol-based porphyrins **3** and **4**.

Thus, *meso-*AB_3_ and *trans*-A_2_B_2_ cardanol-based porphyrins **3** and **4**, were synthesized respectively by acid-catalyzed condensation of compound **1** by statistical reaction with pyrrole and benzaldehyde (*Method 1*) or with *meso*-phenyldipyrrolmethane **2** (*Method 2*), as shown in Scheme 2 in accordance with different reaction protocols [6,7]. The resulting porphyrins **3** and **4**, brown-red sticky solids, very soluble in CHCl_3_ or CH_2_Cl_2_, have been characterized by FT-IR, UV-Vis, ^1^H- and ^13^C-NMR, and MALDI-TOF techniques. Isolated yields and UV-Vis absorption band of compounds **3** and **4** are reported in Table 1.

**Table 1:** Yields and UV-Vis absorption bands of **3**, **4** and their metalloporphyrins **3a–3e**, **4a–4e**.

Compounds	M	Yields %	λ_max_, nm (CHCl_3_)				
			Soret band	Q bands			
**3**	2H	10	419	516	552	590	646
**3a**	Zn	90	424		554	594	
**3b**	Cu	90	416		539		
**3c**	Co	90	411		530		
**3d**	Fe	80	417				
**4**	2H	15	420	517	553	591	647
**4a**	Zn	90	425		554	596	
**4b**	Cu	90	417		540		
**4c**	Co	90	412		530		
**4d**	Fe	80	419				
cardanol-based A_4_-porphyrin	2H	14 [16]	420	518	556	593	649

For instance, the UV-Vis spectrum of **3** showed a Soret band at 419 nm and Q bands at 516, 552, 590 and 646 nm; the UV-Vis spectrum of **4** showed a 1 nm red shift, with a Soret band at 420 nm and Q bands at 517, 553, 591 and 647 nm. A red shift in the Q bands was also observed in the previously reported cardanol-based A_4_-porphyrin [16]. This suggested to us that the number of the substituents in the porphyrin molecule influences the value of the maximum of absorption in the UV-Vis spectra, producing a red shift when the number of substituents is increased. The MALDI-TOF analysis of the metal free porphyins **3** and **4** showed a cluster of signals centered at *m/z* = 958 [M]^+^ and 1,301 [M]^+^, respectively and consistent with the proposed structures.

^1^H-NMR and FT-IR spectra of **3** and **4** were also consistent with the proposed structures. In fact, ^1^H-NMR spectrum of **3** exhibited a multiplet in the 8.82–8.89 ppm range attributable to the eight protons at the β position of the pyrrole moiety, whereas two typical doublets centered at 8.84 and 8.87 ppm for the β position of the pyrrole moiety were observed in **4**, due to its higher symmetry. The aromatic protons, found in the 8.24–6.83 ppm range and the protons of the double bond of the side-chain in the 5.28-5.42 ppm range appear as multiplets, and were similar in both porphyrins **3** and **4**. In the case of **3**, two multiplets corresponding to the protons of the O–CH_2_CH_2_–O system were found in the 5.30–5.40 and 4.59–4.64 ppm range, but in the case of **4** two triplets were found at 4.53 and 4.63 ppm. The triplets centered 2.63 and 2.64 ppm in **3** and **4**, respectively, correspond to the aliphatic protons of the Ar–CH_2_ system, the other aliphatic protons were in the range 0.75–2.12 ppm in both **3** and **4**. NH protons were present as a broad band centered at −2.77 and −2.76 ppm in **3** and **4**, respectively. The FT-IR spectra of porphyrins **3** and **4** showed a weak band at 3,317 cm^−1^, characteristic of the NH vibration, and at 3,006 cm^−1^ attributed to the side-chain vinylic =C–H vibration.

**Scheme 3:**
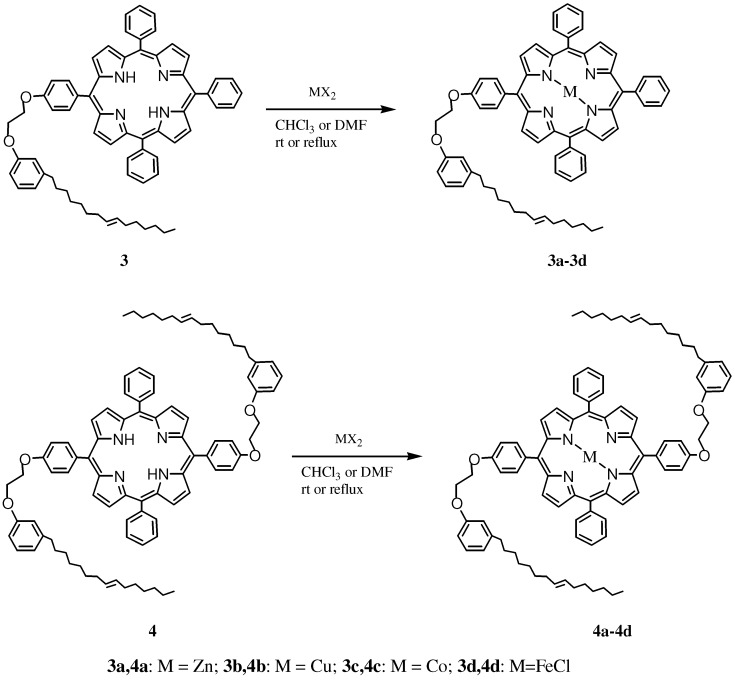
Preparation of the metallo-porphyrins **3a–3d** and **4a–4d**.

Porphyrins **3** and **4** were next used for preparation of the corresponding metallo-derivatives **3a–3d** and **4a–4d** (Scheme 3) in nearly quantitative yields, by reacting them with Zn(OAc)_2_, Co(OAc)_2_·4H_2_O, CuCl_2_, and FeCl_3,_ respectively. FT-IR, UV-Vis, MALDI-TOF and elemental analyses of the metalloporphyrin complexes **3a–3d** and **4a–4d** were consistent with the proposed structures. Yields and UV-Vis absorption bands of **3a–3d** and **4a–4d** are also reported in Table 1.

From the UV-Vis absorption bands it is possible to observe that in the case of metalloporphyrins **3a–3d** and **4a–4d**, the Soret band is only slightly shifted compared to the corresponding metal-free porphyins and the Q bands are reduced to two or at least one because the symmetry of porphyrin ring increases when the hydrogen atoms were replaced by metals.

The IR spectra of **3a–3d** and **4a–4d** were close to those of the corresponding metal-free porphyins **3** and **4**, except for the disappearance of the NH vibration at 3317 cm^−1^. MALDI-TOF mass spectrometry analysis was successfully used for the determination of the molecular weight of the metalloporphyrin complexes **3a–3cd** and **4a–4d** (see Experimental Section). ^1^H and ^13^C-NMR spectra were recorded only in the case of Zn(II) complex **3a** and **4a** because of the paramagnetic effect of the Cu(II), Co(II) and Fe(III) metal ions that hindered the recording of any such spectra.

### 2.2. Preparation of the Cardanol Based Porphyrin/TiO_2_ Composites and Diffuse Reflectance (DR) Spectroscopy Characterization

**Figure 2:**
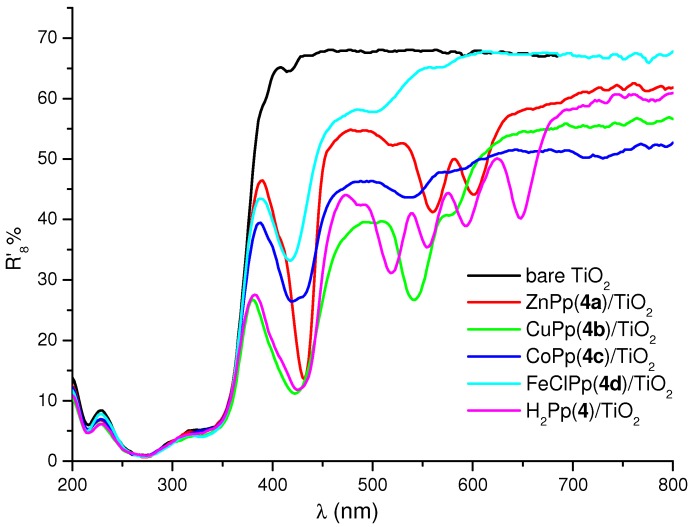
Diffuse reflectance spectra of bare TiO_2_ and differently loaded samples obtained by impregnation of TiO_2_ with H_2_Pp **4** or MPp **4a–4d**.

TiO_2_ composites used as photocatalysts were prepared by impregnating of TiO_2_ with cardanol-based porphyrins according with the procedure reported in the Experimental. Figure 2 shows the diffuse reflectance spectra in air of the bare TiO_2_ as well as of TiO_2_ impregnated with 4.0 µmol of the selected H_2_Pp **4** or MPps [M = Zn (II),Cu(II), Co(II), Fe(III)-Cl] porphyrins **4a–4d** *per gram* of TiO_2_, respectively, recorded in the 200–800 nm range.

It is worth noting that no appreciable shift of the band gap edge of TiO_2_ can be observed for any of the loaded samples. This behaviour was in accord with previously studied metal free and copper [5,10,15,20-tetra(4-tertbutylphenyl)] porphyrins [19].

**Figure 3:**
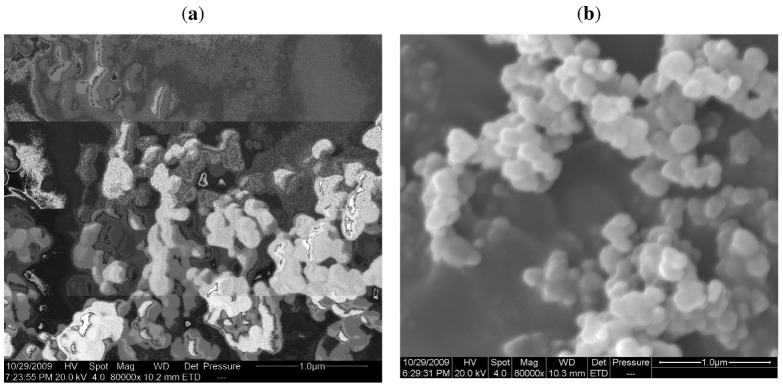
SEM images of bare (**a**) TiO_2_ and (**b**) 4 µmol CuPp (**4a**)/1 g TiO_2_.

Similar behavior was observed for the porphyrins H_2_Pp, **3**, and MPps [M = Zn (II), Cu(II), Co(II), Fe(III)–Cl] **3a–3d**) (spectra not shown in Figure 2 for clarity). Figure 3 shows the SEM pictures of bare TiO_2_ and CuPp (**4b)**/TiO_2_. Basically, the microstructures of the bare TiO_2_ and porphyrin impregnated TiO_2_ composites show common features which are typical regarding this TiO_2_ polymorph. In fact, both kinds of samples seem rather similar, with spherical shaped particles.

### 2.3. Photoreactivity Experiments

A few years ago, we reported that polycrystalline TiO_2_ samples impregnated with differently substituted porphyrins synthesized from commercially available starting materials displayed better photocatalytic activity, in comparison with polycrystalline bare TiO_2_ samples, in the photocatalytic degradation of 4-NP in water [19,20]. In this work, novel cardanol-based composites **3**/TiO_2_, **3a**/TiO_2_-**3d**/TiO_2_ and **4**/TiO_2_, **4a**/TiO_2_-**4d**/TiO_2_, were tested in the photocatalytic degradation of 4-NP.

The efficiency of a photodegradation catalyst has been evaluated by measuring the rate of consumption of 4-NP in a slurry containing a finely dispersed semiconductor, under constant illumination. It can also be noticed that the substrate was degraded using each of the photocatalysts, following pseudo-first-order kinetics. The list of used samples is reported in Table 2, along with the initial reaction rates of 4-NP disappearance as *r*_0_ × 10^9^ (mol L^−1**.**^s^−1^), *r*_0_′ × 10^9^ (mol L^−1**.**^s^−1**.**^m^−2^) and % conversion of 4-NP.

**Figure 4:**
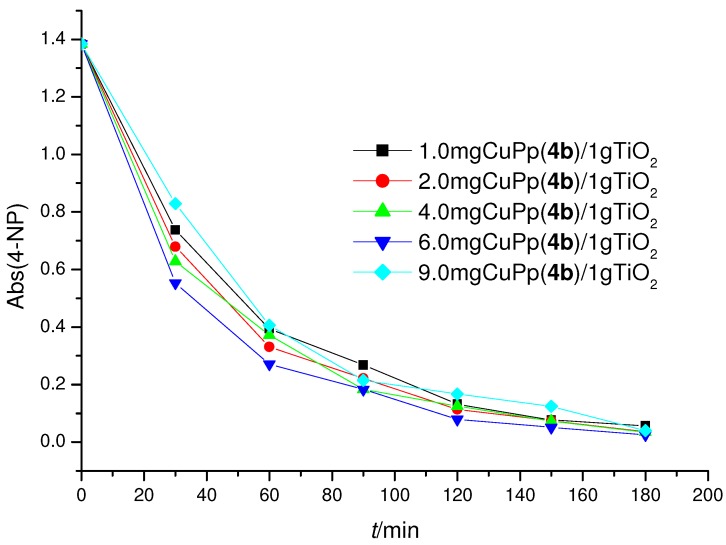
4-NP concentration *vs*. irradiation time using different amounts of CuPp (**4b**)/TiO_2_ photocatalysts.

Figure 4 shows the diminution of 4-NP concentration *vs.* irradiation time using different amounts of CuPp (**4b**)/TiO_2_ photocatalysts. These preliminary investigations were carried out in order to establish which among the differently impregnated photocatalysts exhibited the highest photoactivity.

**Table 2:** List of the samples used together with the initial photoreaction rates, and the conversion (%) 4-NP after 180 min of irradiation time.

Samples ^a^	*r*_0_ × 10^9^	*r*_0_′ × 10^9^	4-NP
	(mol^.^L^−1.^s^−1^)	(mol^.^L^−1.^s^−1.^m^−2^)	(%) ^b^ converted at 180 min
TiO_2_	26.59	33.24	93.5
1.0 µmol CuPp(4b)/TiO_2_	36.36	45.45	95.9
2.0 µmol CuPp(4b)/TiO_2_	39.62	49.52	97.5
4.0 µmol CuPp(4b)/TiO_2_	42.48	53.10	97.4
6.0 µmol CuPp(4b)/TiO_2_	46.70	58.38	98.2
9.0 µmol CuPp(4b)/TiO_2_	31.21	39.01	97.1
6.0 µmol ZnPp(4a)/TiO_2_	33.94	42.42	95.1
6.0 µmol CoPp(4c)/TiO_2_	34.28	42.85	95.4
6.0 µmol FeClPp(4d)/TiO_2_	18.77	23.46	92.8
6.0 µmol H_2_Pp(4)/TiO_2_	22.20	27.75	86.0
4.0 µmol CuPp(3b)/TiO_2_	34.34	42.92	95.8
6.0 µmol CuPp(3b)/TiO_2_	42.03	52.54	97.9
6.6 µmol CuPp(3b)/TiO_2_	41.52	51.90	96.7
6.0 µmol ZnPp(3a)/TiO_2_	33.55	41.94	94.7
6.0 µmol CoPp(3c)/TiO_2_	33.72	42.15	93.9
6.0 µmol FeClPp(3d)/TiO_2_	18.66	23.32	93.3
6.0 µmol H_2_Pp(3)/TiO_2_	21.36	26.70	85.5

^a^ The numbers before the code used for identifying the samples indicate the mg amounts of sensitizer [H_2_Pp(a), H_2_Pp(a), CuPp(a) or CuPp(a)] per gram of TiO_2_; *r*_0_: The initial photoreaction rates per used mass; *r*_0_′: Initial photoreaction rates per used mass and per unit surface area of the catalysts. The BET specific surface areas of all the samples are equal to ca. 8 m^2^**^.^**g^−1^, amount of photocatalyst: 0.1 g/125 mL solution; ^b^ The % conversion of 4-NP was calculated by the following formula C/C_0_ × 100.

It can be seen that the samples impregnated with 6.0-CuPp (**4b)**/TiO_2_ exhibited the highest photoactivity. These results are in accord with those observed by using the sensitizers **3a–3d** as summarized in Table 2.

**Figure 5:**
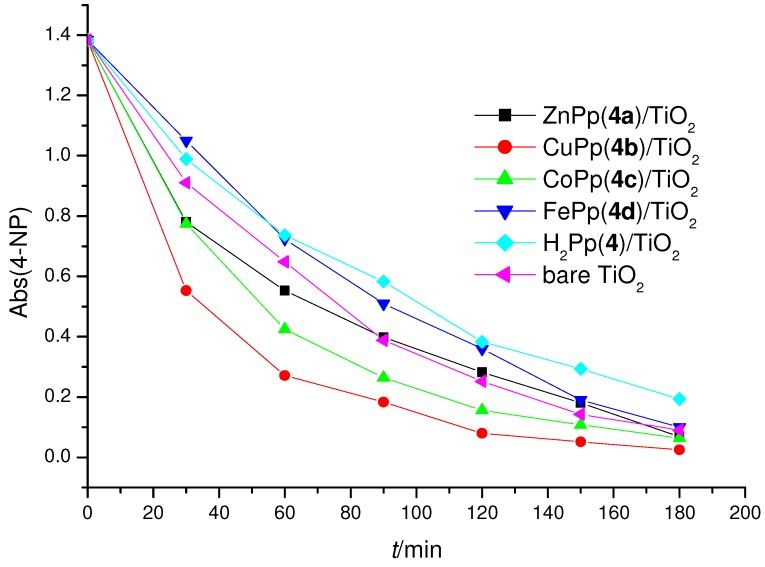
4-NP concentration *vs* irradiation time using 6.0 µmol of H_2_Pp (**4**) or MPps (**4a–4d**) porphyrins/1 g TiO_2_ as photocatalysts.

As shown in the Figure 5, the Cu(II) porphyrin **4b** definitely proved a more effective sensitizer in the photodegradation of 4-NP than other MPp’s (M = Co, Zn) **4a**, **4c**, which have a slight beneficial effect. Interestingly, in contrast with previous experimental evidence [19,20,21], there is a detrimental effect observed for the free-base and Fe(III) porphyrin composites **4**/TiO_2_ and **4d**/TiO_2_ compared with bare TiO_2_ which could be ascribed to the different lamp used as irradiation source.

The photocatalytic activities are also very slightly influenced by the substitutions and the spatial positions of the substitutions of porphyrins. In particular, the composites (**4**, **4a–4d**)/TiO_2_ when used as catalysts show slightly better photocatalytic activities than (**3**, **3a–3d**)/TiO_2_, but they have a similar activity order. All the studied cases gave a conversion of 4-NP higher than 85.5%; in particular, by using the most efficient CuPps/TiO_2_ photocatalysts the measured conversion was close to 98% (Table 2).

**Figure 6:**
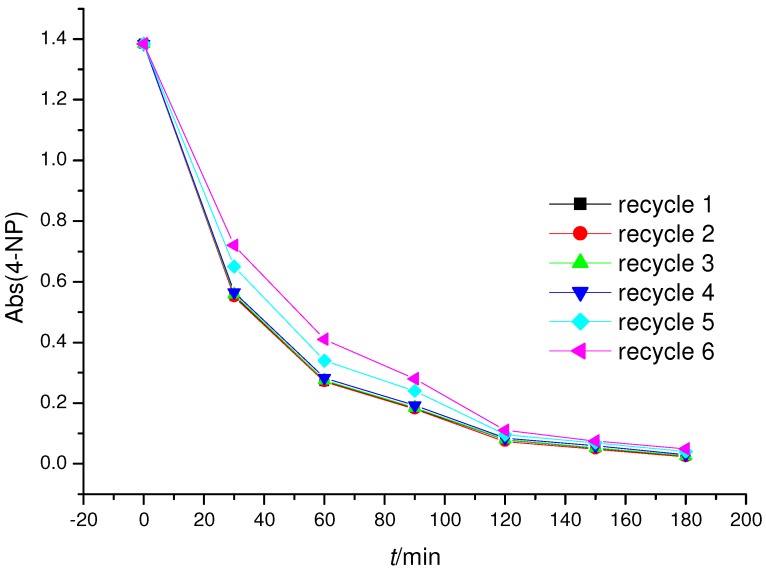
Experiments carried out using recycled 6.0 µmol CuPp (**4b**)/TiO_2_ as the photocatalyst.

Further investigations were carried out in order to establish the photostability of the CuPp **4b** impregnated onto the TiO_2_ surface. Repeated recycling experiments confirmed that this porphyrin supported onto TiO_2_ showed good stability under irradiation conditions and samples continued to maintain good photocatalytic activity after several cycles. Figure 6 shows how the most active photocatalyst, *i.e.*, CuPp (**4b**) TiO_2_ can be recycled six times, after its first use, without significant loss of activity.

Typically, **3b** and **4b**, being effective sensitizers, were insoluble in the water and stable under UV irradiation, and the catalysts **3b**/TiO_2_ and **4b**/TiO_2_ were also reused several times without loss of the activity.

Taking into account the *r*_0_ values reported in the Table 2 of the impregnated MPps were in the following order: CuPp > CoPp *>* ZnPp *>* bare TiO_2_*>* H_2_Pp *>* FePp. The results related to the photo-degradation of 4-NP in an aqueous heterogeneous environment suggest that the Cu(II)-Cu(I) photocatalytic redox cycle plays the main beneficial role for the occurrence of the whole process. In a previous work [20] we demonstrated that Cu(II) could be reduced to Cu(I) [see equation (1)] by electrons of the conduction band of TiO_2_ where additional electrons are injected, due to the presence of the sensitizer:

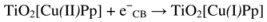
(1)

Despite the complex mechanism of reactions the redox process reported in equation 1 seems to be the key step in the course of which is possible to increase the amounts of OH radicals and superoxide anion responsible of the degradation process of 4-NP [19,20]. Moreover, in the present case, porphyrin sensitizers containing un-saturated chains capable of being oxidized have been used for the first time. Spectroscopic analysis (UV-Vis, FT-IR, *etc.*) carried out in order to check the photostability of the porphyrins used as the sensitizers permitted us to prove the stability of the double bonds contained in the side cardanol chains. In fact, typical spectroscopic signals of double bond of cardanol are still present at the end of each process. This could means that the oxidizing species responsible of the photo degradation processes by oxidative demolition of the 4-NP [19,20] act in water solution far from the composite TiO_2_ surface.

## 3. Experimental

### 3.1. Reagents

Cardanol oil (technical grade) was kindly provided by Oltremare S.p.A. (Bologna, Italy). TiO_2_ (anatase phase, specific surface area 8 m^2^/g), kindly provided by Tioxide Huntsman was dried and crushed to obtain particles with a diameter smaller than 0.1 mm. All other starting materials were purchased from Aldrich Chemical Co and used as received. Silica gel (Merck) was used in the chromatographic separations. Solutions of 4-nitrophenol, used without further purification, were prepared by dissolving the required quantity of 4-NP in water obtained from a New Human Power I water purification system.

### 3.2. Analyses

FT-IR spectra were recorded on a JASCO FT-IR 430 spectrometer. UV-Vis spectra were recorded on a Cary 100scan UV-visible spectrophotometer. ^1^H- and ^13^C-NMR spectra were recorded on a Bruker Avance 400 instrument at room temperature and chemical shifts are reported relative to tetramethylsilane.Laser desorption/ionization time of flight mass spectrometery (LDI-TOF MS) was performed on a Reflex IV spectrometer (Bruker Daltonik, Bremen, Germany), managed by the Flex Control 2.4 software (Bruker Daltonik, Bremen, Germany), equipped with a VSL-337ND nitrogen laser (Laser Science Inc., Franklin, MA, USA) delivering 4 ns pulses with a repetition rate of 5 Hz and an average power of 200 µJ. The laser attenuation setting was typically in the range 40–50. Spectra were obtained in positive ion reflector mode, with 20–17 kV accelerating voltage and 23 kV reflection voltage. External quadratic calibration was performed with a standard mixture ranging from 757.40 to 3147.47 kDa, giving a mass error lower than 15 ppm. One µL of sample solution of CHCl_3_ was spotted on a MTP 384 massive target T (Bruker Daltonik, Bremen, Germany), both in the absence and in the presence (Matrix Assisted LDI-TOF MS) of same volume of alpha-cyano-4-hydroxycinnamic acid (saturated solution in water/acetonitrile/TFA 66.9/33/0.1) as ionization adjuvant, and air-dried. Each spectrum was acquired by 100 to 200 laser shots. Diffuse reflectance (DR) spectra were obtained at room temperature in the wavelength range 200–800 nm using a Shimadzu UV-2401PC spectrophotometer with BaSO_4_ as reference material.

### 3.3. Synthesis

#### 3.3.1. Synthesis of the cardanol based precursors of the porphyrins

*4-[2-(3-(Pentadeca-8-enyl)phenoxy)-ethoxy]-benzaldehyde* (**1**) was synthesized in our laboratory [6,18]; meso-phenyldipyrrolmethane (**2**) was synthesized with the standard procedure in the literature [7].

#### 3.3.2. Synthesis and characterization of *5,10,15-triphenyl-20-mono-[4-(2-(3-pentadec-8-enyl) phenoxy)ethoxy] phenylporphyrin* (**3**)

Compound **3** was obtained by statistical synthesis starting from a 3:1 benzaldehyde-**1** mixture using a procedure similar to that reported in reference [7]. Yield: 10%; ^1^H-NMR (CDCl_3_): δ −2.77 (br, s, 2H), 0.75–0.95 (m, 3H), 1.12–1.42 (m, 16H), 1.58–1.72 (m, 2H), 1.96–2.08 (m, 4H), 2.63 (t, 2H, *J* = 7.6 Hz), 4.49–4.54 (m, 2H), 4.59–4.64 (m, 2H), 5.30–5.40 (m, 2H), 6.83–6.93 (m, 3H), 7.24–7.27 (m, 1H), 7.33 (d, 2H, *J* = 8.6 Hz), 7.71–7.81 (m, 9H), 8.13 (d, 2H, *J* = 8.6 Hz), 8.19–8.24 (m, 6H), 8.82–8.89 (m, 8H); ^13^C-NMR (CDCl_3_): δ 14.6, 23.1, 27.6, 27.7, 29.5, 29.7, 29.7, 29.8, 29.9, 30.1, 30.2, 31.7, 31.9, 32.2, 36.3, 66.9, 67.2, 112.0, 112.9, 113.3, 115.5, 115.7, 120.4, 120.5, 120.6, 121.4, 121.8, 127.1, 128.1, 128.4, 129.7, 129.8, 130.3, 130.4, 135.0, 135.3, 136.0, 142.6, 142.6, 145.3, 155.8, 158.9, 159.1; FTIR (neat), *v*/cm^−1^: 3317, 3006, 2923, 2852, 1598, 1583, 1508, 1470, 1441, 1350, 1244, 1175, 1157, 1109, 1072, 1032, 1001, 979, 966, 932, 909, 876, 845, 800, 731; UV-Vis (CH_2_Cl_2_) λ_max_, nm: 419, 516, 552, 590, 646; MALDI-TOF MS *m/z*: 958 [M]^+^; Molecular weight: 958 amu; Anal. Calc. for C_67_H_66_N_4_O_2_: C, 83.41; H, 7.02; N, 6.91. Found: C, 83.84; H, 6.88; N, 5.84%.

#### 3.3.3. Synthesis and characterization of *5,15-diphenyl-10, 20-di-4-(2-(3-pentadec-8-enyl)phenoxy)ethoxy]phenyl porphyrin* (**4**)

Aldehyde **1** (0.45g, 1 mmol) and *meso*-phenyldipyrrole **2** (0.22g, 1 mmol) in chloroform (150 mL) were stirred at room temperature for 10 min, and then BF_3_·OEt_2_ (3.75 mL of 0.1 M solution in CHCl_3_, 0.375 mmol) was added. The reaction mixture was stirred at room temperature for 24 h, then DDQ (0.17 g in CHCl_3_) was added slowly to the solution with vigorous stirring. Subsequently, the reaction mixture was stirred at room temperature for a further 24 h and then removed the solvent under vacuum. The reaction mixture was passed through a silica gel chromatography column (CH_2_Cl_2_/hexane 6/4 v/v). Yield: 15%; ^1^H-NMR (CDCl_3_): δ −2.76 (br, s, 2H), 0.80–0.95 (m, 6H), 1.16–1.46 (m, 32H), 1.58–1.72 (m, 4H), 1.96–2.12 (m, 8H), 2.64 (t, 4H, *J* = 7.7 Hz), 4.53 (t, 4H, *J* = 4.5Hz), 4.63 (t, 4H, *J* = 4.5 Hz), 5.28–5.42 (m, 4H), 6.83–6.94 (m, 6H), 7.24–7.30 (m, 2H), 7.34 (d, 4H, *J* = 8.7 Hz), 7.72–7.82 (m, 6H), 8.14 (d, 4H, *J* = 8.7 Hz), 8.22 (d, 4H, *J* = 7.4 Hz), 8.84 (d, 4H, *J* = 4.5 Hz), 8.87 (d, 4H, *J* = 4.7 Hz); ^13^C-NMR (CDCl_3_): δ 14.3, 14.6, 23.1, 23.3, 26.0, 26.1, 27.7, 27.7, 29.4, 29.7, 29.7, 29.8, 29.8, 29.8, 29.8, 29.9, 30.1, 30.1, 30.1, 30.1, 30.2, 31.9, 32.0, 32.2, 36.5, 66.9, 67.3, 112.0, 113.3, 115.5, 120.2, 120.3, 120.4, 120.5, 121.8, 127.1, 128.1, 128.4, 128.6, 129.7, 130.3, 130.4, 130.4, 130.6, 135.0, 135.4, 135.4, 136.0, 142.7, 142.7, 145.2, 145.2, 158.9, 159.2; FTIR (neat), *v*/cm^−1^: 3317, 3006, 2923, 2853, 1727, 1602, 1583, 1508, 1454, 1401, 1376, 1350, 1245, 1174, 1158, 1111, 1072, 1001, 980, 966, 932, 907, 876, 844, 801, 733; UV-Vis (CH_2_Cl_2_) λ_max_, nm: 420, 517, 553, 591, 647; MALDI-TOF MS *m/z*: 1303 [M]^+^; Molecular weight: 1303 amu; Anal. Calc. for C_90_H_102_N_4_O_4_: C, 82.61; H, 7.74; N, 4.51. Found: C, 82.95; H, 7.83; N, 4.30%.

#### 3.3.4. General procedure for the synthesis of **3a–3c**, **4a–4c**

Porphyrin **3** (30.0 mg, 0.031 mmol) or **4** (30.0 mg, 0.023 mmol) were dissolved in CHCl_3_ (20 mL). To this solution was added an excess of Zn(CH_3_COO)_2_ (34.0 mg, 0.186 mmol), CuCl_2_ (20.0 mg, 0.186 mmol) or Co(CH_3_COO)_2_·4H_2_O (46.3 mg, 0.186 mmol), and the mixture was stirred at room temperature. The reaction was checked by TLC. After disappearance of **3** or **4**, the solution was filtered and then the solvent was removed under vacuum. The crude product was purified by silica gel chromatography (CHCl_3_/Hexane, 7/3 v/v) to give **3a**, **3b**, **3c**, **4a**, **4b**, **4c** in nearly quantitative yields.

#### 3.3.5. General procedure for synthesis of **3d** and **4d**

Porphyrin **3** (30.0 mg, 0.031 mmol) or **4** (30.0 mg, 0.023 mmol) were dissolved in DMF (15 mL). To this solution, an excess of FeCl_3_ (30.2 mg, 0.186 mmol) was added. The reaction was heated to reflux and monitored by UV/Vis spectroscopy. The metal insertion was completed in 4 h. Then the solvent was removed under vacuum and the residue was purified by silica gel chromatography (CHCl_3_/hexane, 7/3 v/v) to give **3d** and **4d** respectively.

#### Representative data for compounds **3a–3d**, **4a–4d**

*Zn(II)5,10,15-Triphenyl-20-mono-[4-(2-(3-pentadec-8-enyl)phenoxy)ethoxy]phenylporphyrin* (**3a**). Purplish red solid. Yield 95%; ^1^H-NMR (CDCl_3_): δ 0.86–0.90 (m, 3H), 1.26–1.39 (m, 16H), 1.62–1.68 (m, 2H), 1.96–2.01 (m, 4H), 2.63 (t, 2H, *J* = 7.4 Hz), 4.48–4.50 (m, 2H), 4.58–4.61 (m, 2H), 5.33–5.39 (m, 2H), 6.83–6.90 (m, 3H), 7.23–7.27 (m, 1H), 7.32 (d, 2H, *J* = 8.6 Hz), 7.73–7.78 (m, 9H), 8.13 (d, 2H, *J* = 8.6 Hz), 8.22–8.24 (m, 6H,), 8.95–8.99 (m, 8H); ^13^C-NMR (400 MHz, CDCl_3_): δ 14.6, 23.1, 27.6, 27.7, 29.6, 29.7, 29.7, 29.7, 29.8, 30.1, 30.1, 30.2, 31.8, 32.2, 36.4, 68.2, 112.0, 113.2, 115.5, 122.0, 126.9, 127.2, 127.9, 128.0, 128.4, 128.6, 129.7, 130.3, 130.4, 130.4, 130.5, 130.8, 132.3, 132.4, 132.4, 134.9, 135.8, 143.3, 145.2, 145.2, 150.6, 158.5; FTIR (neat), *v*/cm^−1^: 3007, 2924, 2853, 1600, 1584, 1485, 1446, 1378, 1339, 1255, 1157, 1071, 1024, 997, 912, 873, 797, 774, 720, 695; UV-Vis (CHCl_3_) λ_max_, nm: 424, 554, 594; MALDI-TOF MS: an isotopic cluster peaking at *m/z*: 1021 [M]^+^; Molecular weight: 1021 amu; Anal. Calc. for C_67_H_64_N_4_O_2_Zn: C, 78.61; H, 6.14; N, 5.54. Found: C, 78.82; H, 6.27; N, 5.49%.

*Cu(II)5,10,15-triphenyl-20-mono-[4-(2-(3-pentadec-8-enyl)phenoxy)ethoxy]phenylporphyrin* (**3b**). Red solid. Yield: 95%; FTIR (neat), *v*/cm^−1^: 3007, 2924, 2849, 1599, 1583, 1509, 1491, 1442, 1377, 1346, 1245, 1216, 1175, 1158, 1072, 1003, 799, 753, 717; UV-Vis (CHCl_3_) λ_max_, nm: 416, 539; MALDI-TOF MS: An isotopic cluster peaking at *m/z*: 1021 [M]^+^; Molecular weight: 1021 amu; Anal. Calc. for C_67_H_64_N_4_O_2_Cu: C, 78.61; H, 6.14; N, 5.54. Found: C, 78.82; H, 6.27; N, 5.49%.

*Co(II)5,10,15-Triphenyl-20-mono-[4-(2-(3-pentadec-8-enyl)phenoxy)ethoxy]phenylporphyrin* (**3c**). Peach-red solid. Yield: 85%; FTIR (neat), *v*/cm^−1^: 3006, 2924, 2853, 1600, 1583, 1510, 1491, 1448, 1475, 1350, 1283, 1244, 1175, 1158, 1072, 1004, 796, 752, 716, 701; UV-Vis (CHCl_3_) λ_max_, nm: 411, 530; MALDI-TOF MS: An isotopic cluster peaking at *m/z*: 1017 [M]^+^; Molecular weight: 1017 amu; Anal. Calc. for C_67_H_64_N_4_O_2_Co: C, 79.05; H, 6.29; N, 5.51. Found: C, 78.89; H, 6.16; N, 5.64%.

*Fe(III)5,10,15-Triphenyl-20-mono-[4-(2-(3-pentadec-8-enyl)phenoxy)ethoxy]phenylporphyrin chloride* (**3d**). Brown red solid. Yield: 85%; FTIR (neat), *v*/cm^−1^: 3008, 2924, 2853, 1599, 1588, 1485, 1455, 1377, 1340, 1247, 1156, 1072, 1004, 875, 802, 750, 719, 699; UV-Vis (CHCl_3_) λ_max_, nm: 417; MALDI-TOF MS: An isotopic cluster peaking at *m/z*: 1013 [M−Cl−H]^+^; Molecular weight: 1049.5 amu; Anal. Calc. for C_67_H_64_N_4_O_2_FeCl: C, 76.61; H, 6.10; N, 5.33. Found: C, 76.72; H, 6.23; N, 5.54%.

*Zn(II)5,15-Diphenyl-10,20-di-[4-(2-(3-pentadec-8-enyl)phenoxy)ethoxy]phenylporphyrin* (**4a**). Purplish red solid. Yield: 95%; ^1^H-NMR (CDCl_3_): δ 0.82–0.94 (m, 6H), 1.18–1.44 (m, 32H), 1.60–1.72 (m, 4H), 1.93–2.09 (m, 8H), 2.64 (t, 4H, *J* = 7.5 Hz), 4.48–4.55 (m, 4H), 4.59–4.66 (m, 4H), 5.25–5.45 (m, 4H), 6.83–6.94 (m, 6H), 7.24–7.30 (m, 2H), 7.33 (d, 4H, *J* = 7.5Hz), 7.72–7.82 (m, 6H), 8.14 (d, 4H, *J* = 8.3 Hz), 8.23 (d, 4H, *J* = 6.5 Hz), 8.94 (d, 4H, *J* = 4.8 Hz), 8.98 (d, 4H, *J* = 5.1 Hz); ^13^C-NMR (CDCl_3_): δ 14.5, 23.1, 23.2, 26.0, 26.1, 27.6, 27.6, 29.4, 29.7, 29.7, 29.8, 29.8, 29.9, 30.1, 30.1, 30.1, 30.2, 30.2, 31.4, 31.8, 31.8, 32.2, 36.5, 66.9, 67.3, 112.1, 113.2, 115.5, 121.4, 121.8, 126.9, 127.9, 128.4, 129.7, 130.2, 130.3, 130.4, 130.5, 132.3, 132.3, 132.4, 134.8, 135.8, 136.0, 143.3, 145.2, 145.2, 150.6, 150.9, 150.9, 158.8, 159.2; FTIR (neat), *v*/cm^−1^: 3007, 2923, 2852, 1602, 1523, 1509, 1485, 1447, 1373, 1338, 1243, 1174, 1108, 1069, 997, 931, 879, 846, 797, 753, 720, 700; UV-Vis (CHCl_3_) λ_max_, nm: 425, 554, 596; MALDI-TOF MS: An isotopic cluster peaking at *m/z*: 1365 [M]^+^; Molecular weight: 1365 amu; Anal. Calc. for C_90_H_100_N_4_O_4_Zn: C, 79.01; H, 7.54; N, 4.21. Found: C, 79.12; H, 7.33; N, 4.10%.

*Cu(II)5,15-Diphenyl-10,20-di-[4-(2-(3-pentadec-8-enyl)phenoxy)ethoxy]phenylporphyrin* (**4b**). Red solid. Yield: 95%; FTIR (neat), *v*/cm^−1^: 3008, 2925, 2852, 1601, 1583, 1504, 1446, 1377, 1345, 1244, 1215, 1174, 1158, 1072, 1000, 800, 749,701; UV-Vis (CHCl_3_) λ_max_, nm: 417, 540; MALDI-TOF MS: An isotopic cluster peaking at *m/z*: 1365 [M]^+^; Molecular weight: 1365 amu; Anal. Calc. for C_90_H_100_N_4_O_4_Cu: C, 79.12; H, 7.33; N, 4.10. Found: C, 78.92; H, 7.26; N, 4.24%.

*Co(II)5,15-Diphenyl-10,20-di-[4-(2-(3-pentadec-8-enyl)phenoxy)ethoxy]phenylporphyrin* (**4c**). Peach-red solid. Yield: 95%; FTIR (neat), *v*/cm^−1^: 3006, 2924, 2853, 1602, 1583, 1463, 1377, 1351, 1261, 1215, 1175, 1158, 1074, 1005, 799, 755, 701; UV-Vis (CHCl_3_) λ_max_, nm: 412, 530; MALDI-TOF MS: An isotopic cluster peaking at *m/z*: 1361 [M]^+^; Molecular weight: 1361 amu; Anal. Calc. for C_90_H_100_N_4_O_4_Co: C, 79.35; H, 7.35; N, 4.11. Found: C, 79.22; H, 7.31; N, 4.27%.

*Fe(III)5,15-Diphenyl-10,20-di-[4-(2-(3-pentadec-8-enyl)phenoxy)ethoxy]phenylporphyrin chloride* (**4d**). Brown red solid. Yield: 85%; FTIR (neat), *v*/cm^−1^: 3005, 2924, 2853, 1600, 1582, 1509, 1485, 1449, 1376, 1338, 1246, 1158, 1071, 998, 875, 801, 751, 722, 697; UV-Vis (CHCl_3_) λ_max_, nm: 419; MALDI-TOF MS: An isotopic cluster peaking at *m/z*: 1358 [M-Cl]^+^; Molecular weight: 1393.5 amu; Anal. Calc. for C_90_H_100_N_4_O_4_FeCl: C, 77.50; H, 7.18; N, 4.02. Found: C, 77.65; H, 7.23; N, 4.12%.

### 3.4. Preparation of the Cardanol-Based Porphyrin/TiO_2_ Composites

The loaded samples used as photocatalysts for the photoreactivity experiments were prepared by impregnating TiO_2_ with cardanol-based porphyrins. The procedure is as follows: An opportune amount of sensitizer **3a–3d** and **4a–4d** was dissolved in CH_2_Cl_2_ (20 mL) and finely ground TiO_2_ (1 g) was added into this solution. The mixture was stirred for 3–4 h and the solvent removed under vacuum. The resulting composites were marked as **3a**/TiO_2_-**3d**/TiO_2_ and as **4a**/TiO_2_-**4d**/TiO_2_, respectively.

### 3.5. Photo-Reactivity Experiments

Photoreactivity experimenta were carried out in a set-up equipped with a UV lamp (250 W Hg 200 ULTRA lamp), the distance between lamp and the surface of solution is 40 cm with an intensity of 30 W/m^2^. The temperature inside the reactor was maintained at ca. 300 K. The reacting aqueous suspension of 4-nitrophenol (4-NP, 20 mg/L, 125 mL) and catalyst (100 mg) was stirred with a magnetic bar. The initial pH of the suspension was adjusted to 4.0 by the addition of H_2_SO_4_. Air was bubbled into the suspension when switching on the lamp. Samples (3 mL) were withdrawn from the suspension every 30 min during the irradiation. The photocatalysts were separated from the solution by centrifugation and successively filtered through 0.45-µm celluloseacetate membranes (HA, Millipore) before to perform the quantitative determination of 4-NP by measuring its absorption at 316 nm with UV-Vis spectrophotometer. Bare TiO_2_ was also tested for the sake of comparison under the same experimental conditions.

## 4. Conclusions

In this paper we have described the synthesis and characterization of some new cardanol-based porphyrins, 5,10,15-triphenyl-20-mono-[4-(2-(3-pentadec-8-enyl) phenoxy) ethoxy] phenyl porphyrin, (**3**), and 5,15-diphenyl-10,20-di-[4-(2-(3-pentadec-8-enyl)phenoxy)ethoxy]phenyl porphyrin, (**4**), as well as their zinc(II), copper(II) and cobalt(II) metal derivatives, **3a**, **3b**, **3c**, **4a**, **4b** and **4c**. Selected Cu(II) porphyrins used as sensitizers onto TiO_2_ samples showed the best photo-catalytic activity for the photo-degradation of 4-NP in water, compared with the other MPp/TiO_2_ composites.
